# Flexible Switching of Feedback Control Mechanisms Allows for Learning of Different Task Dynamics

**DOI:** 10.1371/journal.pone.0054771

**Published:** 2013-02-06

**Authors:** Olivier White, Jörn Diedrichsen

**Affiliations:** 1 Institut National de la Santé et de la Recherche Médicale, Unit 1093, Cognition, Action and Sensorimotor Plasticity, Dijon, France; 2 Université de Bourgogne, Campus Universitaire, Unité de Formation et de Recherche en Sciences et Techniques des Activités Physiques et Sportives, Dijon, France; 3 Institute of Cognitive Neuroscience, University College London, London, United Kingdom; 4 Wolfson Centre for Clinical and Cognitive Neuroscience, Bangor University, Bangor, United Kingdom; The University of Western Ontario, Canada

## Abstract

To produce skilled movements, the brain flexibly adapts to different task requirements and movement contexts. Two core abilities underlie this flexibility. First, depending on the task, the motor system must rapidly switch the way it produces motor commands and how it corrects movements online, i.e. it switches between different (feedback) control policies. Second, it must also adapt to environmental changes for different tasks separately. Here we show these two abilities are related. In a bimanual movement task, we show that participants can switch on a movement-by-movement basis between two feedback control policies, depending only on a static visual cue. When this cue indicates that the hands control separate objects, reactions to force field perturbations of each arm are purely unilateral. In contrast, when the visual cue indicates a commonly controlled object, reactions are shared across hands. Participants are also able to learn different force fields associated with a visual cue. This is however only the case when the visual cue is associated with different feedback control policies. These results indicate that when the motor system can flexibly switch between different control policies, it is also able to adapt separately to the dynamics of different environmental contexts. In contrast, visual cues that are not associated with different control policies are not effective for learning different task dynamics.

## Introduction

In everyday life, we often use our hands to manipulate objects or tools. This poses a control challenge, because hand and arm movements need to be substantially different when grasping an object with a pair of pliers compared to when grasping the same object directly with hands. Not only must feed-forward commands be different; the motor system must also react to unexpected events, such as a sudden perturbation force on the tool, in a different way. In the context of optimal feedback control theory, both feed-forward and feedback commands can be understood as being generated by a control policy, a mapping between an estimate of the state of the body and the next motor command [1], [2]. Thus, one core skill for the motor system is to retrieve the control policy that is appropriate for a given task context.

A second, related capacity is required in the context of learning. When the motor system encounters a new tool, it needs to be able to learn the new task dynamics associated with this tool in a way that does not generalize so broadly as to interfere with other control policies. In other words, generalization of learned skills should be limited to the correct context. We argue here that the capacity to switch control policies is a primary component of the capacity to learn different task dynamics.

The motor system appears to be highly proficient in changing even very fast feedback control loops based on task context [3], [4], [5], [6]. For example, we have shown that feedback control of bimanual reaching movements depends on the type of object controlled with the two hands [7], [8]. When participants control separate cursors with each hand and one hand is mechanically pushed off its path, corrections are limited to the perturbed hand. In contrast, when the two hands jointly control a single cursor, presented at the spatial midpoint of the hands, perturbations to one arm lead to fast, proprioceptively-driven feedback corrections in both arms. This sharing of online corrections constitutes a better solution to the control problem than separate control, as it reduces effort and signal-dependent noise. In a first set of experiments, we show that the motor system switches without loss on a trial-by-trial basis between these two different feedback control policies using only a static visual cue.

In contrast to its flexibility in switching control policies, the nervous system’s ability to learn different task dynamics in different contexts is often limited [9]. Many studies have investigated this phenomenon using robotic devices to apply velocity-dependent forces perpendicular to the direction of an arm movement. Although participants can adapt to a single force field very quickly [10], learning is much more difficult if the force field changes direction on a trial-by-trial basis. Surprisingly, this continues to be true even when the change in direction is fully predictable using an alternating sequence of perturbations [11], [12] or a predictive visual cue [13]. In other contexts, however, the motor system is quite capable of learning different force field dynamics, for example when the two fields are associated with different tools [14], objects [15], targets [16] or effectors [17], [18].

Here we propose that the critical factor in determining whether simultaneous learning of different dynamics is possible is whether the two contexts were associated with different control policies *before* learning. In the last experiments, participants experienced force fields with different directions that were indicated by a static visual cue. The visual cue also indicated a switch in the task (one vs. two-cursor task), and hence the control policy, or was not related to the task. In support of our hypothesis, we show that a visual cue only favors independent learning of different force fields if it necessitated a switch of control policy before learning.

## Methods

### Participants

All experimental and consent procedures were approved by the ethics committee of the School of Psychology at Bangor University (United Kingdom). A total of 39 right-handed volunteers (Exp 1: n = 9, Exp 2: n = 10, Exp 3: n = 20) aged 18 to 44 years (mean age 24.4 years, SD = 7 years) participated in the experiments. All participants gave their written informed consent to participate in this study. All had normal or corrected to normal vision and did not report any motor disabilities. They were naïve as to the purpose of the experiments and were debriefed after the experimental session. Participants were reimbursed for their time with a per-session payment of £6.

### Apparatus and Stimuli

**Figure 1:**
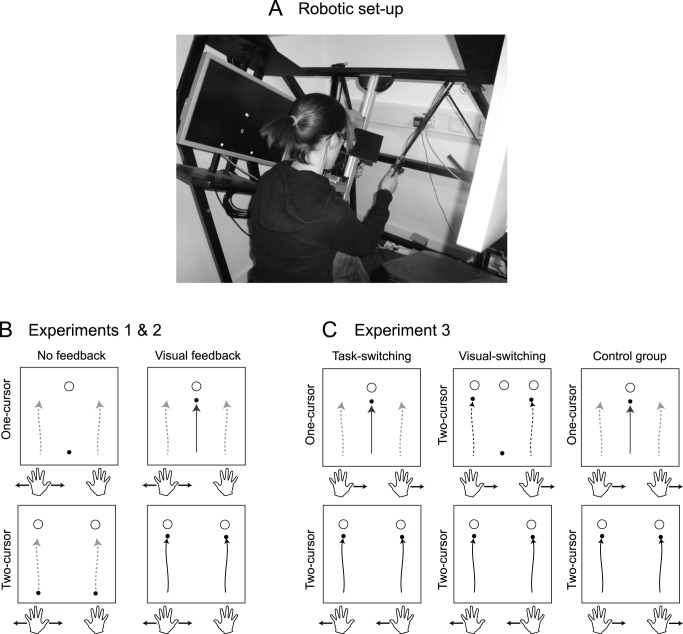
General procedures and experimental conditions. (A) Picture showing the experimental setup. (B) In Experiments 1 & 2, in the one-cursor condition (upper row), one target (empty circle) and one cursor (black disk) were presented; in the two-cursor condition (lower row), two targets and two cursors were displayed. In some trials, visual feedback about cursor position was provided throughout the movement (solid black line(s)); in others, only the initial and final positions of the cursor(s) were displayed. Light gray dashed arrows denote hand positions. In Experiment 1, a velocity-dependent force field was applied to one hand, in either the left or the right direction (shown only for the left hand). (C) In Experiment 3, the force field was presented to both hands and switched direction randomly across trials. In the task-switching group, the cursor condition predicted force field direction. The visual-switching group always performed the two-cursor task, but a visual cue indicated force field direction (the presence or absence of the middle target indicated the direction of the force field). The control group experienced a force field in the same direction in all trials.

Participants were comfortably seated in front of a virtual environment equipment with the head on a chin rest (Fig. 1a). A horizontal crossbar stabilized the upper body and minimized interaction torques between left and right arm movements. They made 10 cm (Exp 1 and 2) or 12 cm (Exp 3) reaching movements while holding on to a robotic device with each hand (Phantom 3.0, SensAble Technologies, USA). Movements were performed in the natural reaching space in an upward-forward direction, involving shoulder and elbow movements, with the elbow pointing downwards. Participants looked into two mirrors that were mounted at 90 degrees to each other, such that they viewed one LCD screen with the right eye and one LCD screen with the left eye. This stereo display was calibrated such that the physical locations of the robotic arms were consistent with the visual disparity information. Throughout the experiment, 3-dimensional grey spheres (6 mm diameter cursors) indicated participants’ hand positions.

### General Procedure

To start a trial, participants moved the two robot handles, which were visually indicated as two cursors, into starting spheres (8 mm diameter), displayed 6 cm to the left and right of the midline at chest height. A static visual cue then indicated whether participants performed the one- or two-cursor task. In the one-cursor condition, a single target (8 mm diameter) was presented at body midline, and a single cursor was presented at the spatial average position of the two hands. In the two-cursor condition, two targets were presented 10 cm (12 cm in Exp 3) above each starting sphere. Importantly, in this cueing phase the cursor(s) was (were) frozen on the screen and did not move with any potential small hand movements.

Participants were instructed to reach the target(s) by moving both hands rapidly upwards. Movement start was detected online when both hands exceeded a speed of 35 mm/s. In trials with visual feedback, the cursors (dark circles in Fig. 1b,c) were displayed during the movement at the current hand position. In trials without visual feedback, the cursor(s) disappeared at movement onset and reappeared at movement offset, defined by the moment when both hand speeds fell below 15 mm/s for at least 40 ms. Movement times of less than 800 ms with a spatial accuracy of better than 4 mm were rewarded with a target explosion and one point. These movement times and spatial thresholds were adjusted after each block to keep participants at a level of approximately 60% correct trials.

On trials with a force field, the robotic arm generated a force in the horizontal direction (perpendicular to the movement trajectory) (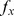) that was proportional to the forward velocity (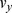) of the hand following 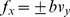 with 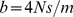.

### Experiment 1: Blocked Task-switching

Participants performed two sessions separated by at least one day. Each session consisted of eight blocks of 64 trials. The cursor condition alternated between 1 and 2 cursors every 16 trials. On each trial, a leftward or rightward force field was presented to one of the hands with equal probability. The sideways force was proportional to the upward velocity along the movement plane. Visual feedback of the moving cursor(s) was provided on half the trials. The trials were randomly intermixed, such that participants could not predict the side or direction of the force field and whether visual feedback would be presented.

### Experiment 2: Random Task-switching

The apparatus, instructions and task were identical to those used in Exp. 1. Participants performed first two training blocks of 24 trials with visual feedback in both the one- and two-cursor conditions. In half the practice trials, a force-field was applied to one of the hands in a random direction. In the following eight blocks containing 64 trials each, the cursor condition changed randomly from trial to trial. Therefore, in contrast to Exp. 1 where the switching occurred in regular intervals, participants needed to rely on the static visual cue to know whether they would perform the one- or two-cursor task. As in Exp 1, feedback of the cursor(s) during the movement was randomly withdrawn on half the trials. Furthermore, on half the trials, a force field was applied to one of the hands. The perturbed hand and the direction of the force field were varied randomly between trials.

### Experiment 3: Force Field Learning during Random Task-switching

Experiment 3 was designed to determine whether switching between different control policies would be associated with the ability to learn different forward dynamics. For this purpose, we studied three groups. The *task-switching* group (N = 8) switched randomly between the one- and two-cursor tasks (Fig. 1c, “Task-switching” column). To prevent longer sequences of one task, we introduced the constraint that every task was presented maximally twice in a row. As in the other experiments, the static presentation of one or two target(s) and one or two cursor(s) before movement onset indicated task condition. One task condition was always associated with a leftward force field on both hands, the other with a rightward force field on both hands. The task-to-force-field assignment was counterbalanced between participants.

The *visual-switching* (N = 8) group always performed the two-cursor task. As with the task-switching group, the randomly switching force field was associated with a visual cue that indicated whether the force field on both hands would be rightward or leftward. This static cue was either two targets and two cursors, or a middle target and a middle cursor. Because participants always performed the two-cursor task, the lateral targets were always presented. Therefore, the visual cue associated with one force-field direction consisted in three targets and the cue associated with the other direction consisted in two targets (see Fig. 1c, “Visual switching” column). Both groups were instructed that the visual cue would be predictive of the direction of the force field. Thus, the only difference between the task-switching and visual-switching groups was whether the predictive visual cue was also associated with a different controlled object.

To control for non-predictive learning processes, such as strategic increases in stiffness across the task, both groups performed the experiment under two conditions. In the predictable condition, the direction of the force field on both hands was linked to the visual cue as described above. In the unpredictable condition, the force field switched independently of the visual cue. The sequence of the two conditions was counterbalanced between participants.

We also included a control group (N = 4), which performed the experiment twice, once with a constant leftward force field on both hands, once with a constant rightward force field on both hands (Fig. 1c, “Control group” column). As the task-switching group, they randomly alternated between the one- and two-cursor tasks, again triggered by the visual cue. This group provided a baseline of how well the force fields could be learned when no interference was present.

Each participant started with a practice block of 56 trials in the two-cursor condition (all groups) and the one-cursor condition (task-switching and control groups). They then performed two sessions of four blocks of 56 trials (224 trials per session). Each session consisted of four trials of baseline without a force field, 196 trials with a randomly switching (task- and visual-switching groups) or constant force field (control group), and 24 trials with no force field to allow for washout. The 196 trials of force field were separated into 4 phases of 49 trials. Eight randomly chosen trials from each phase were force channel trials, designed to probe force-field learning. On these trials, the robot simulated a position-dependent spring (1000 N/m) in the horizontal direction, which pushed the hands onto a straight trajectory. The force participants exerted against the channel served as a direct measurement of the learned expectation of a force field [19], [20]. Online feedback about hand positions was provided during all trials of Experiment 3.

### Data Analysis

**Figure 2:**
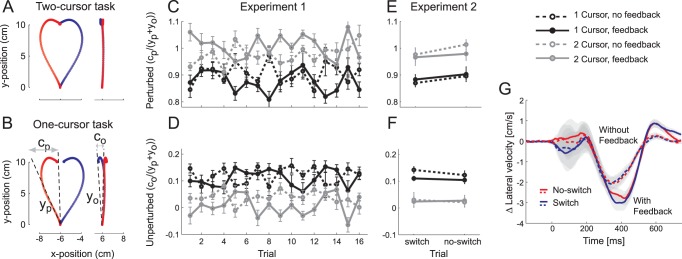
Perfect task-dependent switching of feedback control in Experiments 1 and 2. (A, B) Average position trace of the perturbed hand (left) and the unperturbed hand (right) for trials with a leftward (red) or rightward (blue) force field. We measured the initial perturbation angle of the perturbed hand (y_p_) and unperturbed hand (y_o_) and correction by the perturbed (c_p_) and the unperturbed (c_o_) hands. Both hands shared the correction in the one-cursor task but only the perturbed hand corrected in the two-cursor task. (C,D) Correction gains for perturbed (c_p_/(y_p_+y_o_)) and unperturbed (c_o_/(y_p_+y_o_)) hands as a function of trials after a task switch (y is defined as y_p_+y_o_). For the one-cursor task, correction gains decreased on the perturbed and increased on the unperturbed hand. All results are shown averaged across left and right hands. (E,F) Correction gains for perturbed and unperturbed hands for Experiment 2, as a function of whether the task switched on the trial or not. (G) Difference in the horizontal velocity of the unperturbed hand on trials with a leftward or rightward force field on the perturbed hand for the one-cursor task only. The initial correction is not influenced by the presence of visual feedback, and is identical for switch and no-switch trials. Later phases of the feedback response depend on the visual feedback. Error bars and shaded areas are between participants SE.

Position and forces were recorded with a sampling rate of 200 Hz. The initial direction error of each movement was defined as the angular deviation from the straight direction towards the target, 160 ms after movement onset. Note that movement start for offline analysis was calculated with a slightly more sensitive threshold than that used for online visual feedback. In this case, movement start was detected when movement velocity exceeded 3 cm/s for at least 40 ms. In Experiments 1 and 2, we determined the size of the correction performed by the perturbed and the unperturbed hands by comparing hand-angle at movement end to the initial direction error (Fig. 2b). For each trial, we normalized the size of feedback correction (c) by dividing it by the size of the signed initial error summed across the perturbed and unperturbed hands (y_p_+y_o_). The normalized feedback gains were submitted to three-way repeated-measures ANOVA to assess the effects of task switching (switch vs. no-switch trial), cursor and feedback conditions. In Experiment 3, we used the initial error direction and the lateral force exerted against the channel at peak velocity (occurring on average 162 ms after movement start) as a measure of feedback correction. Here, the force field was applied to both hands. Therefore, we averaged these variables across hands.

## Results

### Experiments 1 and 2: Switching between Different Control Policies

In Experiments 1 and 2, we tested how well participants switched between different feedback control policies. On each trial, participants performed one of two tasks. In the two-cursor task, participants viewed two spatial targets and moved two cursors (displayed at the position of each hand) to these targets with a fast, upward arm movement (Fig. 2a). In the one-cursor task, participants viewed a single target and moved a single cursor (displayed at the spatial midpoint of the hands) to that target. This condition introduced redundancy; it was no longer necessary to move both hands straight upwards. Rather, one hand could deviate to the side, as long as the other compensated for the deviation.

Feed-forward motor commands were similar across conditions. The movement times (723 ms), the initial direction of the hands on unperturbed trials (0.09 deg) were not significantly different between the one- and two-cursor tasks (t(8) = −1.93, p = .10; t(8) = .34, p = .746). Also the stiffness of the arm, as measured by the size of the initial perturbation caused by a left- or rightward force field did not differ between conditions (on average 17.1 deg, t(8) = .58, p = .577). Interestingly, however, feedback corrections differed between task conditions. In the two-cursor task, only the perturbed hand performed the feedback correction (Fig. 2a). In the one-cursor task, the other hand corrected in a direction that helped to restore the cursor and the perturbed hand correspondingly corrected less (Fig. 2b).

The focus of Experiment 1 was to determine how fast participants could switch between these two control domains. In this experiment, participants switched every 16 trials between the one- and two-cursor conditions. We were especially interested in examining how visual cues contributed to this flexibility. We therefore compared no-feedback trials, in which participants only viewed a static visual cue (one/two targets and one/two cursors) before the start of the movement, but did not see the cursor(s) while they moved, with full-feedback trials in which they received continuous visual feedback.

To quantify the distribution of feedback correction, we calculated the difference between the initial and final movement angles for the perturbed hand (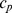, Fig. 2b) and the unperturbed hand (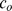). We then expressed the correction of each hand relative to the size of initial angular error summed across hands. Thus, a correction gain of 1 would imply that a hand fully corrected for the common angular error, whereas a correction gain of 0.5 for both the perturbed and unperturbed hands would imply symmetric corrections.

Figure 2c, d shows the magnitude of the corrections for perturbed and unperturbed hands as a function of the trial number after the switch. To test for changes in these correction gains we conducted a task×feedback×trial repeated measures ANOVA. In the two-cursor task, corrections were almost entirely performed by the perturbed hand (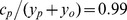), with minimal contribution of the unperturbed hand (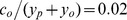). However, in the one-cursor task, the unperturbed hand increased its contribution (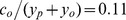) and the perturbed hand corrected less (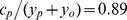). The effect of task was significant for both perturbed (F(1,8) = 49.2, p<.001) and unperturbed hands (F(1,8) = 75.89, p<.001).

Visual feedback during a trial accentuated task differences. If participants received visual feedback, the correction gains for the perturbed hand were higher in the two-cursor and lower in the one-cursor task compared to the no feedback condition, F(1,8) = 30.6, p<.001.

Importantly, however, the task-dependent modulation of the correction gain was already present on the first trial after the switch. The difference between tasks was significant on the first trial, even considering only trials without online feedback about the cursor-position(s) (t(8) = 3.09, p = .015 for the perturbed and t(8) = −3.56, p = .007 for the unperturbed hand). After the first trial, correction gains did not change further. The task×trial interaction was not significant for either the perturbed (F(15,120) = 1.23, p = .258), or unperturbed hands (F(15,120) = 1.19, p = .290).

In sum, the results of Experiment 1 clearly show that participants switched between different feedback control policies without the need of any “warm up” or visual feedback about the controlled object. Furthermore, the degree of the distribution of the feedback correction in this switching experiment was comparable to those found when the two tasks were performed in separate sessions [8], [7].

In Experiment 2, we tested whether participants could switch control policies as easily when the task condition changed randomly on a trial-by-trial basis, rather than across blocks. For half the trials, the task condition remained the same as that on the previous trial; for the other half, it switched, again indicated by a static visual cue. As in Exp. 1, the correction gain of the perturbed hand decreased from 0.99 in the two-cursor condition to 0.88 in the one-cursor condition, F(1,9) = 62.84, p<.001. The correction gain on the unperturbed hand increased from 0.02 to 0.12, F(1,9) = 40.82, p<.001.

Visual feedback during a trial led to larger differences in correction gains for the one- and two-cursor tasks for perturbed relative to unperturbed hands (Fig. 2e, f). The difference in gain was 0.12 for visual feedback but only 0.08 without visual feedback (F(1,9) = 15.85, p = .004). For the unperturbed hand, this difference was not significant (0.11 with and 0.08 without visual feedback, F(1,9) = 2.06, p = .189).

As before, the difference between task conditions did not change as a function of whether participants switched task conditions, or performed the same task as on the previous trial. The task×trial, and the task×feedback×trial interactions were non-significant, for the correction gain of both perturbed and unperturbed hands (all F(1,9)<1.62, p>.239).

Finally, we considered the possibility that switching between control policies would modify the speed, rather than the size of correction. We therefore analyzed the lateral velocity profile of the unperturbed hand in the one-cursor task, depending on whether participants switched between tasks or not, and depending on the presence of visual feedback. For each condition, we calculated the difference in the lateral velocity of the unperturbed hand between trials on which the other hand experienced a rightward or leftward force field. The average difference trace (Fig. 2 g) indicates that the correction in the one-cursor condition emerged at 200 ms, independent of visual feedback. This shows proprioceptive information drives the onset of the coordinative response. Although the later part of the correction was larger when visual feedback was present, there was no difference in correction latency between trials on which the task switched and trials on which the task did not switch.

Therefore, Experiment 2 showed that a static visual cue is sufficient to allow participants to optimally distribute the correction across hands, even when the task switched randomly on a trial-by-trial basis. Both the size and the time course of the correction of the unperturbed hand were the same, whether participants switched control policies on that trial, or whether they repeated the same control policy of the last trial.

### Experiment 3: Adapting to Different Force Fields

We predicted that, if the motor system could effortlessly switch between different feedback control policies, it should also be able to learn different task dynamics – or different feed-forward control – with these control modes. Participants should therefore learn opposing force fields when they are consistently associated with either the one- or two-cursor task. We additionally predicted that a single visual cue would not be sufficient to learn two different force fields if it were not connected to a switch in control policy, even if participants received explicit instruction about the nature of the cue.

Therefore, in Experiment 3 we studied three groups of participants (Fig. 1b). Two groups experienced a force field that switched its direction randomly across trials. For the task-switching group, one force field was associated with the two- and the other with the one-cursor task. The presentation of one or two target(s) and cursor(s) before movement onset indicated both task condition and force field direction. The visual-switching group performed only the two-cursor task, with force-field direction indicated by the presence or absence of the middle target. To control for learning of non-predictive ways of dealing with the force field (e.g., increases in limb stiffness; [21]), both task- and visual switching groups performed under two conditions. In the predictable condition, force field changed direction systematically with the visual cue (and task). In the unpredictable condition, the direction of the force field was unrelated to the visual cue. Finally, we added a control group, which switched between the one- and two-cursor tasks, but experienced a force field with a constant direction.

**Figure 3:**
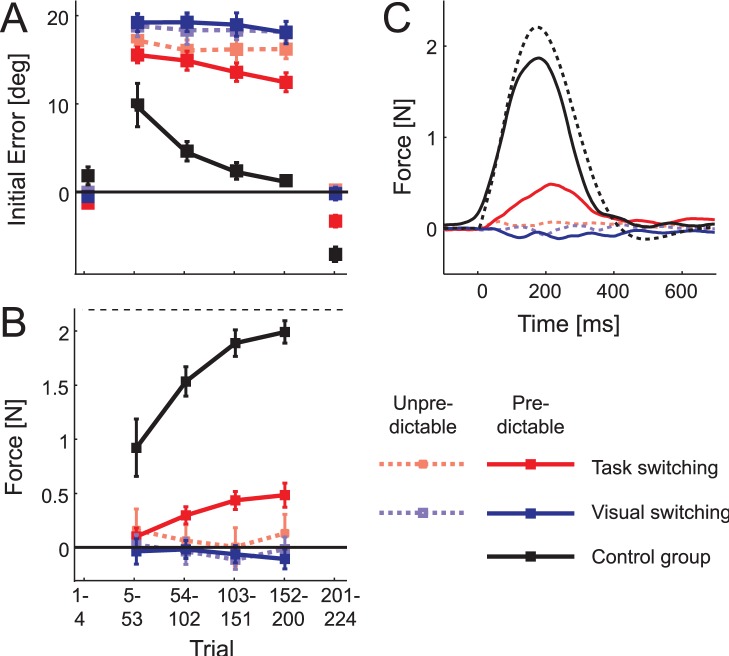
Results of Experiment 3. (A) Angular error at peak velocity for the task-switching (red), visual-switching (blue) and control group (black) as a function of trial. The force field was present from trial 5 to 200. Results averaged across participants, hands and force field directions. Error bars are between participants SE. Positive errors occurred in the direction of the force field. (B) Force at peak velocity for force-channel trials. Positive forces resist the expected force field direction. Error bars are between participants SE. (C) Temporal profiles of forces produced along the channel for the three groups. The dashed line shows the average velocity-dependent force experienced on force field trials.

Figure 3a shows the initial angular error for each group under both conditions. All groups showed an increase in the initial error immediately after force field introduction. The control group (black) reduced this error quickly. To assess whether participants in the other two groups showed any evidence of learning, we compared the predictable and unpredictable conditions. For the visual-switching group (blue), there was no significant effect of predictability, F(1,7) = 0.73, p = .422, whereas for the task-switching group (red) the effect of predictability was significant, F(1,7) = 41.74, p<.001. In a mixed ANOVA, the group×predictability interaction was significant (F(1,7) = 18.45, p<.001). Thus, only the task-switching group showed learning for the predictable condition, amounting to a 23.6% (SD = 14.3%) reduction in initial error.

The aftereffect, a deviation of the initial movement trajectory in the opposite direction of the force field after its withdrawal, corroborated this result. Only the control and task-switching groups, but not the visual switching group, showed a significant aftereffect in the predictable condition, t(8) = 6.2, p<.001. In a direct comparison between the task and visual-switching groups, the group×predictability interaction was again significant, F(1,14) = 15.99, p<.001.

A direct measure of predictive compensation for force fields can be obtained by constraining arm movements with a stiff clamp or force channel around a straight trajectory (see methods). If the motor system anticipates a force field, it pushes into the wall of the channel to counteract the force. As expected, on these control trials, the control group pressed against the channel with a force that closely matched the size of the force they experienced on normal trials (Fig. 3b, dashed line). However, of the experimental groups, only the task-switching group showed a significant learning-related force increase in the predictable condition.

Was ability of the task-switching group to learn due to the fact that these participants already executed movements of different length or direction in the one- and two-cursor task at the beginning of learning? The redundancy in the one-cursor task allows outward or inward movements of the hands, whereas straight movements are required in the two-cursor task. To test this, we compared the initial direction of the hand trajectories at 200 ms after onsets for the 8 participants of the task-switching group. We did not find a significant difference, neither for the left hand (t(7) = .4, p = .689), nor for the right hand (t(7) = -.2, p = .821). We also verified that participants adopted similar initial movement angles between cursor conditions for each hand considered separately. The unperturbed feedforward kinematics in these two conditions was highly overlapping. Importantly, the absolute difference in angle did not correlate with the amount of aftereffect shown in the predictable condition (r = .33, p = .296 for left and r = .01, p = .967 for right hands) – even participants that showed no differences between hands appeared to show a learning effect. Finally, the movement amplitude was also identical across cursor conditions (left hand: t(7) = −2.3, p = .055; right hand: t(7) = 1.7, p = .138). Therefore, the control policy for the left and right hands only differed in the type of feedback corrections, but not in their feed-forward command.

Furthermore, the temporal shape of the force channel response for the control group and for task-switching group matched the velocity-dependence of the originally experienced force field under predictable conditions (Fig. 3c). If the learning exhibited in the task-switching group were due to movement re-planning in Euclidian space (i.e., aiming to the left or the right of the targets), the profile would have shown a different shape, with a force pushing against the channel in the end, rather than the middle of the movement [22]. Thus, our results indicate that participants truly learned to compensate for opposing forward dynamics, as long as the direction of the force field linked to both a different controlled object and a different feedback control policy before learning commenced.

## Discussion

In our experiments, we examined the motor system’s ability to switch between feedback control strategies using a visual cue. In the two-cursor task, feedback control was independent across the hands, with only the perturbed hand correcting for the trajectory deviation. In the one-cursor task, feedback control was dependent, and both hands corrected for perturbations delivered to one hand. Participants switched effortlessly between these two control policies, such that they were fully established on the first trial; repetition of task condition did not lead to further improvements, and distribution of feedback corrections was comparable to that found when the tasks are performed in isolation [7], [8]. Participants switched control policies based on a static visual cue only, without the need for direct experience in controlling the cursor. The immediate switching is especially remarkable, because independent feedback control of the two hands - the preferred mode of control in the two-cursor task - would be sufficient to solve the one-cursor task as well. Thus, switching of control must constitute an automatic and relatively effortless process.

The difficulty in learning two opposing force fields simultaneously has attracted much attention and the nature of the cues that allow or don’t allow learning of separate dynamics has been debated extensively. Some contextual cues, such as color [13], sequence of movements [11], [12] or the shape of the robotic handle [23] do not appear to be able to avoid the catastrophic interference between the two force fields. In contrast, when participants experience different force fields in the context of different tasks, partially independent learning occurs. For example, force field learning while holding on to a robot only partially generalizes to free reaching movements [17], [14]. Force fields in opposite directions can be learned when one force direction is associated with bimanual movements and another with unimanual movements [18], or when the two are associated with different spatial targets [16]. Furthermore, partly separate force field can be learned when one force field is associated with a rigid bimanual object and another is associated with independent hand movements [15]. We show here that participants can learn to associate force fields in opposite directions with two different feedback control policies. The same visual cues, when not connected to a switch in control policy, did not provide a sufficient basis for learning different force fields. Importantly, the feed-forward command in these two situations was identical [16].

Based on these results, we propose that it is only possible to learn different movement dynamics if the two contexts involved at least partially different control policies before learning begins. This allows the motor system to adjust and optimize the control policies independently. In support of this hypothesis, we show that a visual stimulus allows for independent learning only if it is associated with the activation of different feedback control policies and each policy can be adapted to a separate force field.

We believe that most of the literature on how people learn force fields in opposite directions supports this model. Only cues that have implications for action control before force field learning begins can support learning of separate dynamics [17], [14], [18], [15], [16]. Cues that are irrelevant to the main control of the task before force field introduction are harder to learn. One notable exception to this rule is the claim that randomly switching force fields cued by a color or spatial cue can be learned, as long as the cue and force field change randomly [24]. However, our results are at odds with this finding. Participants who experienced visual switching only (Experiment 3) did not show any measurable learning for random switches in force direction over the course of 195 trials. This is despite the fact that the cue was clearly visible in the center of the display and that all participants were instructed at the beginning of each predictable block that the force field direction was linked to the presence or absence of this central cue. We therefore believe that their failure to learn was not caused by an inability to perceive the cue, but rather to the fact that the visual cue was not a priori connected to a switch in control policy (i.e. it was task-irrelevant).

Our results also show that simultaneously learning force fields in opposite directions was still more difficult than learning force fields in the same direction [16], [18], [17]. This implies that the neural representations of the two movement contexts partly overlap. Interestingly, the two feedback control policies in our task were not fully task-dependent. If the feedback control policies had been fully re-optimized based on the task goal, then the burden of correction should have been distributed evenly across the hands in the two-cursor task. However, the unperturbed hand only made 10–15% of the total correction. These results indicate that the control policies are partly task-dependent (and separate for the one- and two-cursor tasks), and partly task-independent (independent control for the two hands, regardless of task). It is tempting to speculate that this common control structure is also responsible for the partial interference in force field learning.

If separate dynamics can only be learned if there are already separate control policies for the two situations, how can the motor system learn to adapt to a completely novel task or situation? In the framework proposed here, this is only possible if the motor system learns to associate the two situations with different control policies. Results indicate that such a bifurcation in learning, i.e., the development of two separate control policies where there was previously only one, may be a very slow process [13]. However, once cues have been associated with different control policies, the same cues can then serve as a basis for faster learning of separate dynamics [25]. For example, different force fields associated with color cues are learned more quickly, if these color cues were experienced in combination with different spatial cues and force fields in opposite directions before learning.

In sum, our results show that participants switch to the optimal feedback control policy flexibly based on a static visual cue, even if it is not strictly necessary for goal achievement, and even if the feed-forward behavior of these control policies is nearly identical. We show that having two different control policies then enables the nervous system to associate different dynamics with two different tasks and to adapt these control policies separately. Thus, the motor system is only blind to those cues that do not help it optimize task control.

## References

[1] Todorov E, Jordan MI (2002) Optimal feedback control as a theory of motor coordination. Nat Neurosci 5: 1226–35. 12404008 10.1038/nn963

[2] Diedrichsen J, Shadmehr R, Ivry RB (2010) The coordination of movement: optimal feedback control and beyond. Trends Cogn Sci 14: 31–39. 20005767 10.1016/j.tics.2009.11.004 PMC4350769

[3] Marsden CD, Merton PA, Morton HB (1981) Human postural responses. Brain 104: 513–534. 7272713 10.1093/brain/104.3.513

[4] Rothwell JC, Traub MM, Marsden CD (1980) Influence of voluntary intent on the human long-latency stretch reflex. Nature 286: 496–498. 7402329 10.1038/286496a0

[5] Pruszynski JA, Kurtzer I, Scott SH (2008) Rapid motor responses are appropriately tuned to the metrics of a visuospatial task. J Neurophysiol 100: 224–238. 18463184 10.1152/jn.90262.2008

[6] Mutha PK, Sainburg RL (2009) Shared bimanual tasks elicit bimanual reflexes during movement. J Neurophysiol 102: 3142–3155. 19793874 10.1152/jn.91335.2008 PMC2804413

[7] Diedrichsen J (2007) Optimal task-dependent changes of bimanual feedback control and adaptation. Curr Biol 17: 1675–9. 17900901 10.1016/j.cub.2007.08.051 PMC2230536

[8] Diedrichsen J, Dowling N (2009) Bimanual coordination as task-dependent linear control policies. Hum Mov Sci 28: 334–347. 19131136 10.1016/j.humov.2008.10.003

[9] Gandolfo F, Mussa-Ivaldi FA, Bizzi E (1996) Motor learning by field approximation. Proc Natl Acad Sci U S A 93: 3843–6. 8632977 10.1073/pnas.93.9.3843 PMC39446

[10] Shadmehr R, Mussa-Ivaldi FA (1994) Adaptive representation of dynamics during learning of a motor task. J Neurosci 14: 3208–24. 8182467 10.1523/JNEUROSCI.14-05-03208.1994 PMC6577492

[11] Conditt MA, Gandolfo F, Mussa-Ivaldi FA (1997) The motor system does not learn the dynamics of the arm by rote memorization of past experience. J Neurophysiol 78: 554–60. 9242306 10.1152/jn.1997.78.1.554

[12] Wainscott SK, Donchin O, Shadmehr R (2005) Internal models and contextual cues: encoding serial order and direction of movement. J Neurophysiol 93: 786–800. 15385598 10.1152/jn.00240.2004

[13] Shadmehr R (2004) Generalization as a behavioral window to the neural mechanisms of learning internal models. Hum Mov Sci 23: 543–568. 15589621 10.1016/j.humov.2004.04.003 PMC2722915

[14] Kluzik J, Diedrichsen J, Shadmehr R, Bastian AJ (2008) Reach adaptation: what determines whether we learn an internal model of the tool or adapt the model of our arm? J Neurophysiol 100: 1455–1464. 18596187 10.1152/jn.90334.2008 PMC2544452

[15] Howard IS, Ingram JN, Wolpert DM (2008) Composition and decomposition in bimanual dynamic learning. J Neurosci 28: 10531–10540. 18923029 10.1523/JNEUROSCI.3473-08.2008 PMC2637175

[16] Hirashima M, Nozaki D (2012) Distinct motor plans form and retrieve distinct motor memories for physically identical movements. Curr Biol 22: 432–436. 22326201 10.1016/j.cub.2012.01.042

[17] Cothros N, Wong JD, Gribble PL (2006) Are there distinct neural representations of object and limb dynamics? Exp Brain Res 173: 689–697. 16525798 10.1007/s00221-006-0411-0

[18] Nozaki D, Kurtzer I, Scott SH (2006) Limited transfer of learning between unimanual and bimanual skills within the same limb. Nat Neurosci 9: 1364–1366. 17028583 10.1038/nn1785

[19] Smith MA, Ghazizadeh A, Shadmehr R (2006) Interacting adaptive processes with different timescales underlie short-term motor learning. PLoS Biol 4: e179. 16700627 10.1371/journal.pbio.0040179 PMC1463025

[20] Scheidt RA, Dingwell JB, Mussa-Ivaldi FA (2001) Learning to move amid uncertainty. J Neurophysiol 86: 971–85. 11495965 10.1152/jn.2001.86.2.971

[21] Burdet E, Osu R, Franklin DW, Milner TE, Kawato M (2001) The central nervous system stabilizes unstable dynamics by learning optimal impedance. Nature 414: 446–9. 11719805 10.1038/35106566

[22] Diedrichsen J, White O, Newman D, Lally N (2010) Use-dependent and error-based learning of motor behaviors. J Neurosci 30: 5159–5166. 20392938 10.1523/JNEUROSCI.5406-09.2010 PMC6632748

[23] Cothros N, Wong J, Gribble PL (2008) Distinct haptic cues do not reduce interference when learning to reach in multiple force fields. PLoS One 3: e1990. 18431477 10.1371/journal.pone.0001990 PMC2291555

[24] Osu R, Hirai S, Yoshioka T, Kawato M (2004) Random presentation enables subjects to adapt to two opposing forces on the hand. Nat Neurosci 7: 111–2. 14745452 10.1038/nn1184

[25] Haruno M, Wolpert DM, Kawato M (2001) Mosaic model for sensorimotor learning and control. Neural Comput 13: 2201–2220. 11570996 10.1162/089976601750541778

